# Additive manufacturing in compact high-gain wideband antennas operating in mm-wave frequencies

**DOI:** 10.1038/s41598-023-38247-x

**Published:** 2023-07-07

**Authors:** Álvaro F. Vaquero, Alejandro Rebollo, Manuel Arrebola

**Affiliations:** 1 Department of Electrical Engineering, Group of Signal Theory and Communications, Universidad de Oviedo, 33203 Gijón, Spain; 2 Instituto de Telecomunicações, Instituto Superior Técnico, 1049-001 Lisbon, Portugal

**Keywords:** Electrical and electronic engineering, Electronic and spintronic devices

## Abstract

A wideband dual-reflector 3D-printed antenna is proposed to operate in the mm-Wave band. The design is based on a Cassegrain reflector optics but including a dielectric piece for merging the feeding system and the support structure of the subreflector. The operational principle of this antenna is presented, as well as the design parameters. Then, a prototype to operate at Ka-band is manufactured combining a 3D-printed technique using PLA as printable material and a spray to coating the antenna, providing a low-cost affordable solution. The different pieces of the antenna are evaluated, and the antenna is also measured in a spherical compact range. An excellent agreement between simulations and measurements is obtained, resulting in a $48.2\%$ of operational bandwidth. These results validate the use of coating procedures and the design technique at these demanding frequencies. Its operation shows a stable gain in the entire Ka-band (including $28$ and $39 \mathrm{GHz}$), which makes the antenna as a suitable light, low-cost, and broadband solution for mm-Wave applications.

## Introduction

Wireless technologies have increased their interest throughout the last decade owing to applications such as microwave imaging (MWI)^1^, radio frequency identification (RFID) or, more recently, wireless power transfer (WPT)^2^ or the Internet of Things (IoT) framework. However, the boost of wireless communications has been reached with the development of the next generation of mobile communications, namely the current fifth generation (5G) or the so-called *beyond* 5G (B5G) and 6G. These generations demand high energy efficiencies together with high data rate systems to enhance the capacity of communications. Those new specifications require to use large bandwidth or multiple bands, and these three generations intend to use frequencies within the (sub)millimeter band of the spectrum. Several regions, such as Europe, EEUU, or Japan have booked bands centered at 28 and 39 GHz (FR2), or even plan to work in higher frequencies up to 200 GHz, to provide high-speed wireless cellular networks^3^.

In this line, space industry has increased their interest in the development of new solutions based on Low and Medium Earth Orbit satellite constellations to provide 5G global broadband telecommunication service^4,5^. These constellations use small platforms, small-geo satellites for geostationary orbits or mega-constellations of CubeSat^6,7^ in the lower orbit to provide the telecommunication services. As it is well-known space industry demand the highest performance on their devices, pushing the research community to do their best. Thus, spaceborne antennas are characterized for being always on the edge of technology, reaching the tightest performance in terms of beam shaping, efficiency, or reliability^8^.

Satellite communications usually require high-gain antennas, being parabolic reflector the most popular solution^9,10^. In its classical configuration the reflector is illuminated by a primary feed, which needs a feed chain system (orthomode transducers OMTS, diplexers, filters, among others.). However, there are more complex configurations based on dual-reflector topologies, being the most popular the Cassegrain. In this case, the primary feed illuminates the subreflector surface, whose reflection provides the incident field onto the main reflector. This configuration typically improves the gain or radiation efficiency, as well as the radiation performance, such as sidelobe level (SLL), ohmic losses or the noise figure when are compared with single reflectors. The main drawback is the need of supporting structures to hold the subreflector, which increase the blockage losses^11^. Different structures have been proposed in the literature to obtain self-supported subreflectors, being the most common approach the hat-feed reflectors^12–14^. These solutions provide a good trade-off between compact structures and aperture efficiency due to the corrugations used in the metallic hat.

Additive manufacturing (AM) has brought a revolution to many engineering areas, having a deep impact on industry. AM should not be only about changing manufacturing process or taking the advantages of new materials. AM is a matter of enabling powerful new concepts or designs which are not affordable by traditional manufacturing, and it can have a noticeable impact on spaceborne antennas. It is applicable to several materials, ranging from polymers or composites to ceramics, or even metals. It fits well for reaching complex design in single-piece (monolithic) structures, which decrease the weight and volume of devices^15^.

AM has been also applied to reflector antennas^16–18^, namely, to manufacture a dielectric skeleton of the reflector surface using any of the classical 3D techniques, such as SLA or FDM. Then, the skeleton is metallized using vacuum metallization, conductive coating, or electroplating. A further step was introduced in^19^, which presents a dual-reflector antenna with a novel dielectric self-supported subreflector in Cassegrain configuration. This work proposes an innovative technique to avoid supporting struts taking AM as key factor to manufacture the antenna.

This work presents a dual-reflector Cassegrain at Ka-band as a broadband high gain solution to operate between $28$ to $39 \mathrm{GHz}$ (the entire Ka-band). Then, the antenna is prototyped with an AM technique together with a metal spray coating to obtain the conductor surface. A secondary goal is to validate these two techniques and low-cost materials in the Ka-band as a low-cost and easy-manufacturing technique. The antenna is measured in the anechoic chamber to evaluate the performance of the antenna, obtaining a good concordance between simulations and measurements. The antenna exhibits high gain, broadband, and similar performance in the entire Ka-band, validating the materials and design technique at millimeter frequency bands.

## Antenna optics: Cassegrain configuration

### Antenna description

**Figure 1:**
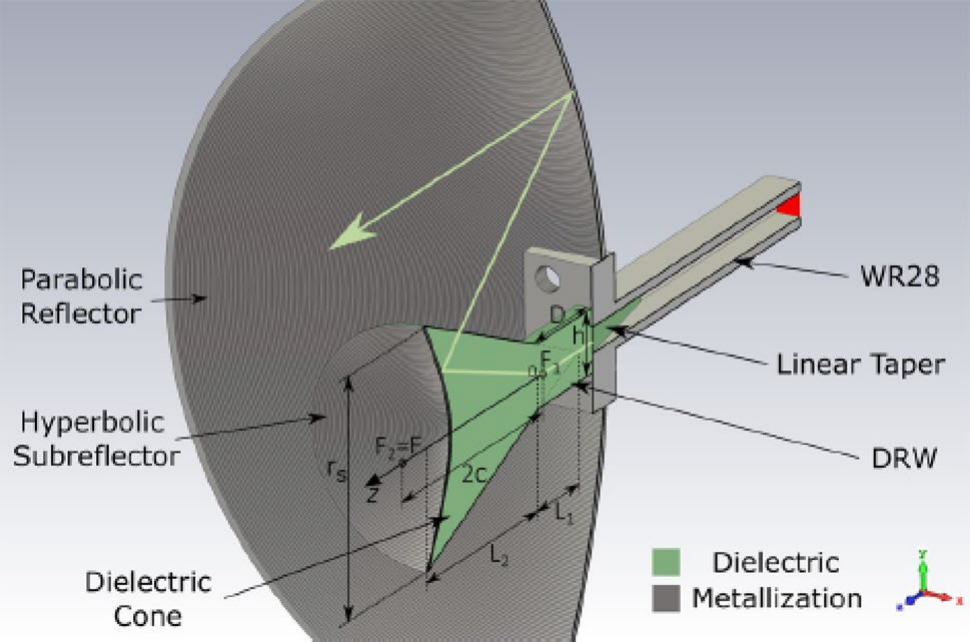
Side view of the proposed antenna, where the design parameters are also represented.

The proposed Cassegrain antenna is made up of a main parabolic reflector, a hyperbolic structure that behaves as a subreflector, and a primary feed as Fig. 1 depicts. According to this configuration, the main reflector has a focal point ($F$) and the subreflector foci (${F}_{1}$ and ${F}_{2}$). Whether the phase center of the primary feed is placed in the focal point ${F}_{1}$, the wave scattered by the hyperboloid subreflector comes apparently from ${F}_{2}$. Then, if the focal point $F$ of the main paraboloid is placed at ${F}_{2}$, the main reflector is focused.

On this basis, the feed of the proposed antenna is based on a standard waveguide WR28 that works as a feed for the antenna as well as supporting structure. It is connected to a Dielectric Rectangular Waveguide (DRW) by an H-plane linear taper. The DRW is gradually widened in a conical shape as Fig. 1 shows. The end of the cone is modified to obtain an axial symmetry hyperbolic surface, which is lately metalized, to reach the hyperboloid subreflector. Therefore, the wave travels through the DRW and the cone to finally be reflected in the hyperboloid and it illuminates the main reflector. Owing to the parabolic geometry of the main reflector, the plane wave is created at the antenna aperture^11^.

### Design procedure

The details of the design process of each element of the self-supported primary feed and subreflector are further described in^19^. Two important factors must be underlined here. First, the focal point ${F}_{1}$ of the hyperbolic subreflector must be in the phase center of the primary feed, which is the DRW. Otherwise, the spherical wavefront that illuminates the main reflector would not be properly formed. This point is placed close to the virtual vertex of the dielectric cone. Second, the cone and the subreflector must be designed so that the dielectric-air interface is as perpendicular as possible to the rays coming from the subreflector reflection. This fact would minimize the refraction produced at the surface of the interface for the reflected field. Otherwise, the refraction would produce a significant variation in the direction of the rays and the spherical wave which illuminates the main paraboloid would be strongly distorted, resulting in antenna defocusing.

**Figure 2:**
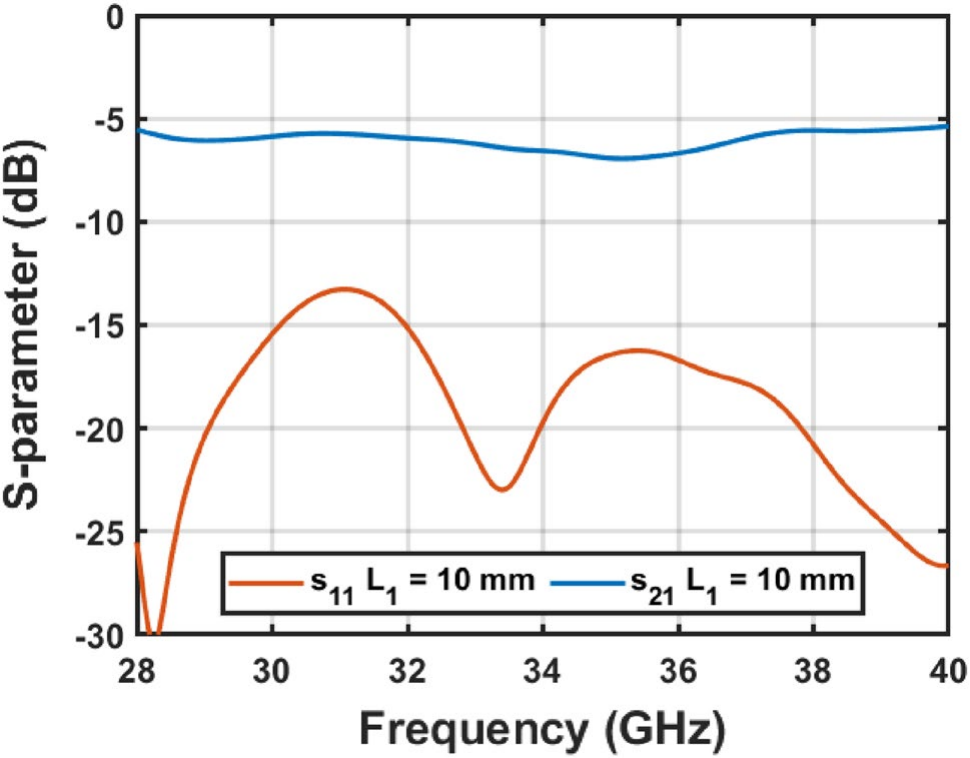
Simulated S-parameters of the DRW plus $H$-taper within the desired band considering the selected length of the DRW: ${L}_{1}=10 \mathrm{mm}$.

The resulting antenna optics achieves a well-focused reflector and subreflector, whose outgoing rays are parallel at the antenna aperture despite the dielectric used for propagating the wave. The design parameters, depicted in Fig. 1, take the values summarized in Table 1 to achieve the beforementioned behavior. Note that, the dielectric used in this antenna is PLA which has a relative high loss^20^, so that the length ${L}_{1}$ is selected to avoid the propagation of evanescent modes but maximizing the transmission power. Figure 2 shows the S-parameters for three different lengths, being the shortest the one used to carry out the design. The DRW is simulated together with the $H$-taper in a full-wave simulation using CST Microwave Studio^21^. For the shortest length the DRW plus taper is matched within the whole desired band, nearly reaching and ${s}_{11}$ better than $-15 \mathrm{dB}$. The ${s}_{21}$ is between $- 5$ and $- 6 \mathrm{dB}$, which might be considered low. However, they are not because of the design but the material as the ${s}_{11}$ shows.

**Table 1:** Geometry of the proposed antenna.

Subreflector			
$\mathrm{b }(\mathrm{mm})$	$10.7$	${\mathrm{L}}_{1} \left(\mathrm{mm}\right)$	$10.4$
$\mathrm{c }(\mathrm{mm})$	$12.9$	${\mathrm{L}}_{2} \left(\mathrm{mm}\right)$	$24.6$
${\mathrm{r}}_{\mathrm{s}} (\mathrm{mm})$	$16.1$	$\mathrm{\alpha }(^\circ )$	$24.6$
$\mathrm{h }(\mathrm{mm})$	$9.6$	$\mathrm{D }\left(\mathrm{mm}\right)$	$13.9$
Reflector			
Focal length $(\mathrm{mm})$	42.2	Diameter $(\mathrm{mm})$	$107.1$

The antenna is designed at the lower frequency $(28 \mathrm{GHz})$, but it should work in the entire Ka-band. Despite not designing the antenna considering the whole band, the working principle used in the antenna definition and design is based on ray tracing and the antenna geometry, thus the antenna should provide broadband performance, limited mainly by the WR28 standard waveguide fundamental mode bandwidth.

### Performance evaluation

**Figure 3:**
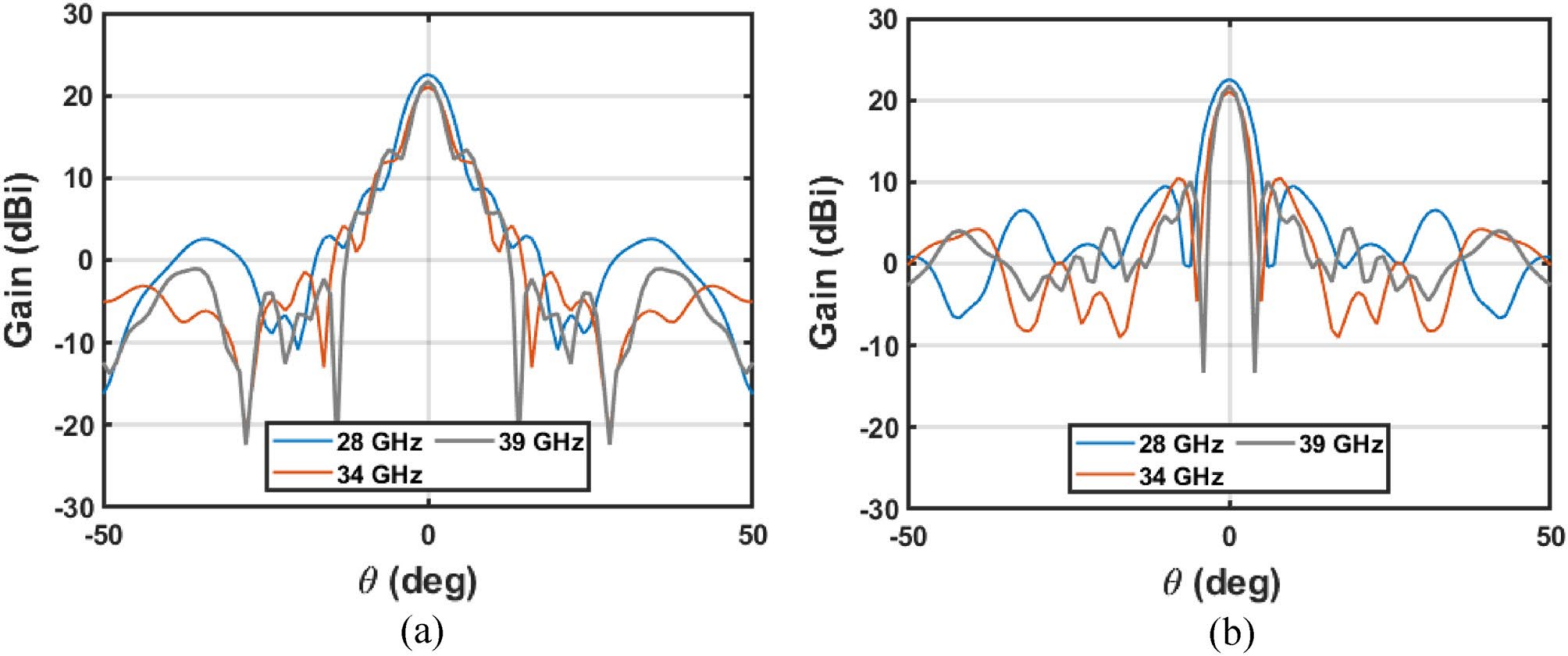
Simulated gain at $28$, $34$ and $39 \mathrm{GHz}$ in the main planes (**a**) $\phi =0^\circ$ (**b**) $\phi =90^\circ$.

The antenna optics of Table 1 is full-wave simulated in CST Microwave Studio to evaluate the gain at three Ka-band frequencies: $$28, 34$$ and $$39 \mathrm{GHz}$$; which are the extreme frequencies and an intermediate one. A dielectric material (PLA) with $${\epsilon }_{r}=2.75$$ and $$\mathrm{tan}\delta =0.015@60\mathrm{GHz}$$^20^ is used for the DRW, cone, and main paraboloid. The metallic surfaces, paraboloid main reflector, hyperboloid subreflector and feeding waveguide are defined as PEC. The planes $$\phi =0^\circ$$ and $$\phi =90^\circ$$ ($$H$$- and $$E$$- plane, respectively) are shown in Fig. 3 for the three frequencies. The gain at $$28 \mathrm{GHz}$$ is $$22.57 \mathrm{dBi}$$, a side lobe level (SLL) of $$13.8 \mathrm{dB}$$ and, a cross-polar level ($$CP/XP)$$ of $$21.63 \mathrm{dB}$$. The antenna at $$39 \mathrm{GHz}$$ reaches a gain of $$21.70 \mathrm{dB}$$, a SLL of $$11.65 \mathrm{dB}$$, and a $$CP/XP$$ of $$18.56 \mathrm{dB}$$. The $$CP/XP$$ is evaluated at $$\phi =45^\circ$$. As shown in the figures, the behavior of the antenna is stable in Ka band and just a slight variation in the simulated parameters is obtained within the Ka-band, as shown at $$28, 34$$ and $$39 \mathrm{GHz}$$.

**Figure 4:**
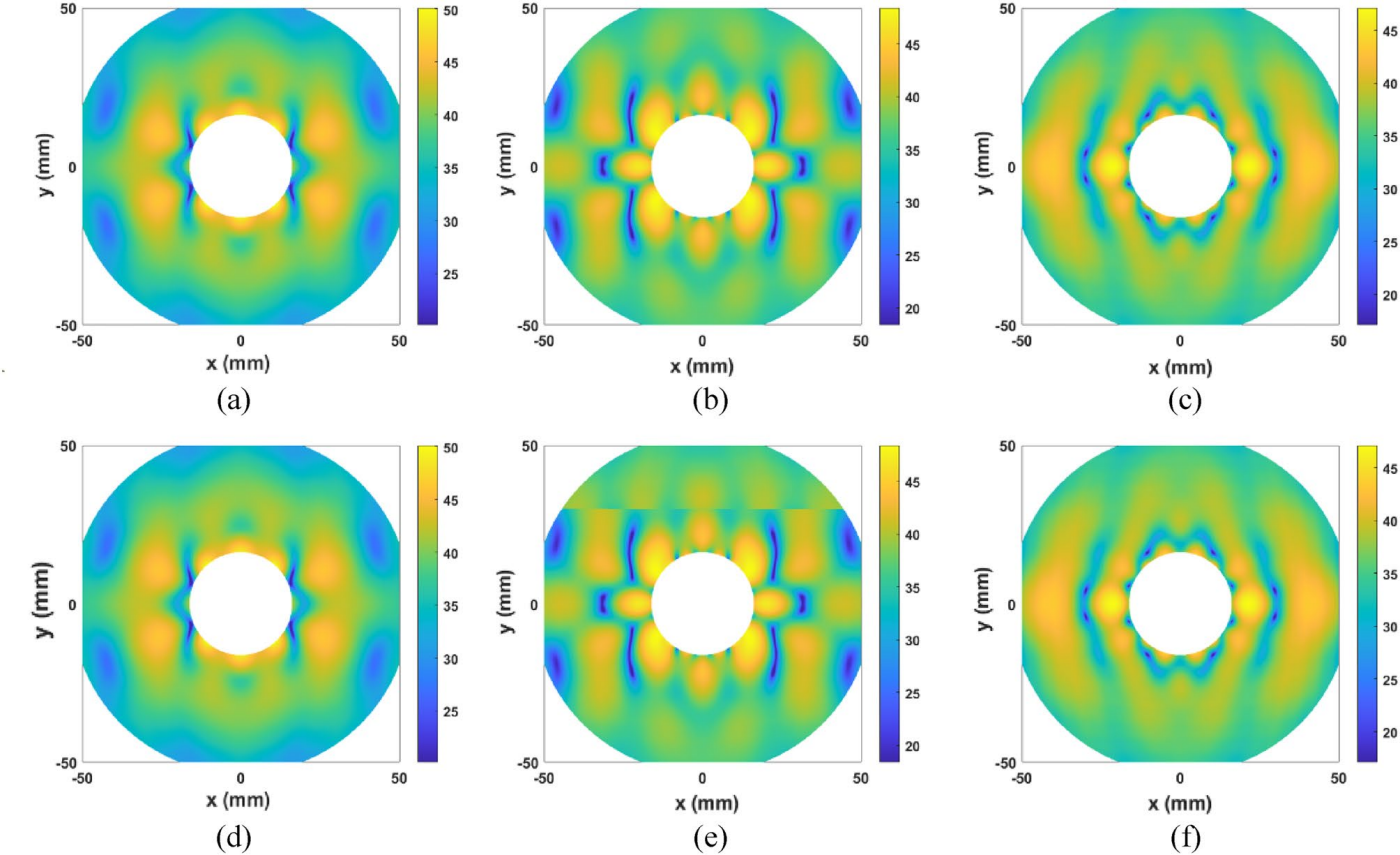
Simulated electric field at the chordal plane of the main reflector (**a**–**c**) considering a PEC as subreflector metallization and (**d**–**f**) a roughness of $0.1 \mathrm{mm}$ in the subreflector metallization at several frequencies (**a**,**d**) $28 \mathrm{GHz}$, (**b**,**e**) $34 \mathrm{GHz}$ and (**c**,**f**) $39 \mathrm{GHz}$.

**Figure 5:**
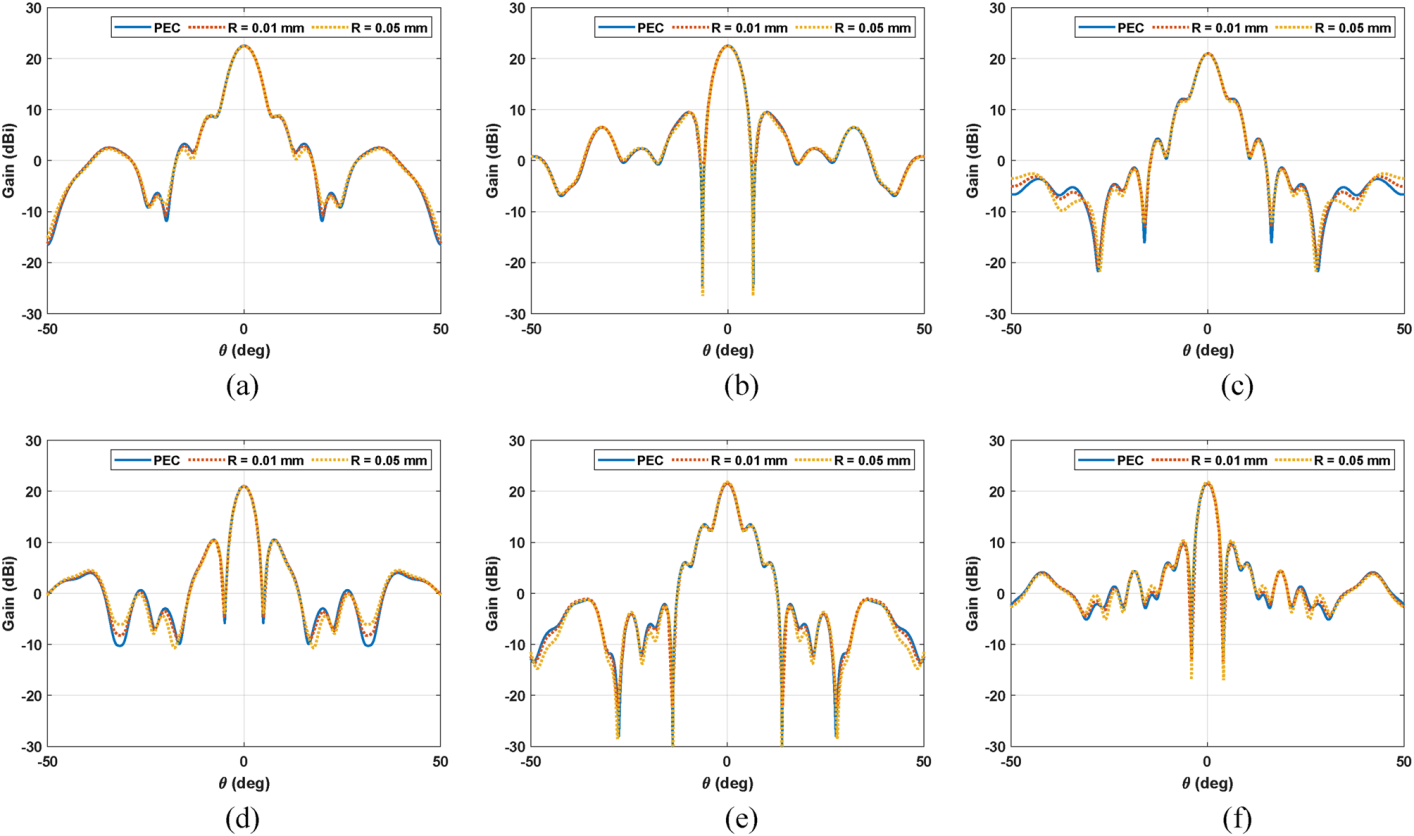
Simulated gain considering PEC and roughness ($0.01$ and $0.05 \mathrm{mm}$) at (**a**,**d**) $28 \mathrm{GHz}$, (**b**,**e**) $34 \mathrm{GHz}$ and (**c**,**f**) $39 \mathrm{GHz}$ in the main planes (**a**–**c**) $\phi =0^\circ$ (**d**–**f**) $\phi =90^\circ$.

The effect of the roughness has been also evaluated in full-wave simulations. To do so, two new simulations are carried out considering $0.01 \mathrm{mm}$ of roughness in the metallization. First, the main reflector is removed and the radiated electric field at its chordal plane is evaluated in order to analyze the effect of the subreflector metallization roughness into the illumination provided by the feeding subsystem (feed and subreflector). Figure 4 compares this case with having a Perfect Electrical Conductor (PEC) as subreflector at several frequencies within the desired bandwidth. These results show that the impact of the roughness is quite low, and the illumination remains almost the same as the case of a PEC. Second, the whole antenna is again full wave simulated considering the $0.01$ mm of roughness in both subreflector and main reflector metallization. For this analysis the radiation pattern is computed at $28, 34$ and $39 \mathrm{GHz}$. Figure 5 shows the comparison with the PEC case, which are the same results previously shown in Fig. 3. The radiation pattern remains the same and the roughness only modifies slightly some nulls or widen the beamwidth. However, the gain is the same as having a PEC. These results might be expected since the accuracy of the 3D-printer is $0.1 \mathrm{mm}$ for each layer and the metallization layers are quite uniform. Moreover, the roughness is short enough in terms of $\lambda$, therefore the scattering of the roughness is quite small to have an impact on the antenna performance and the mismatch produces in the interface dielectric-metallization-air enhances the reflection.

## Experimental validation

### Fabrication

**Figure 6:**
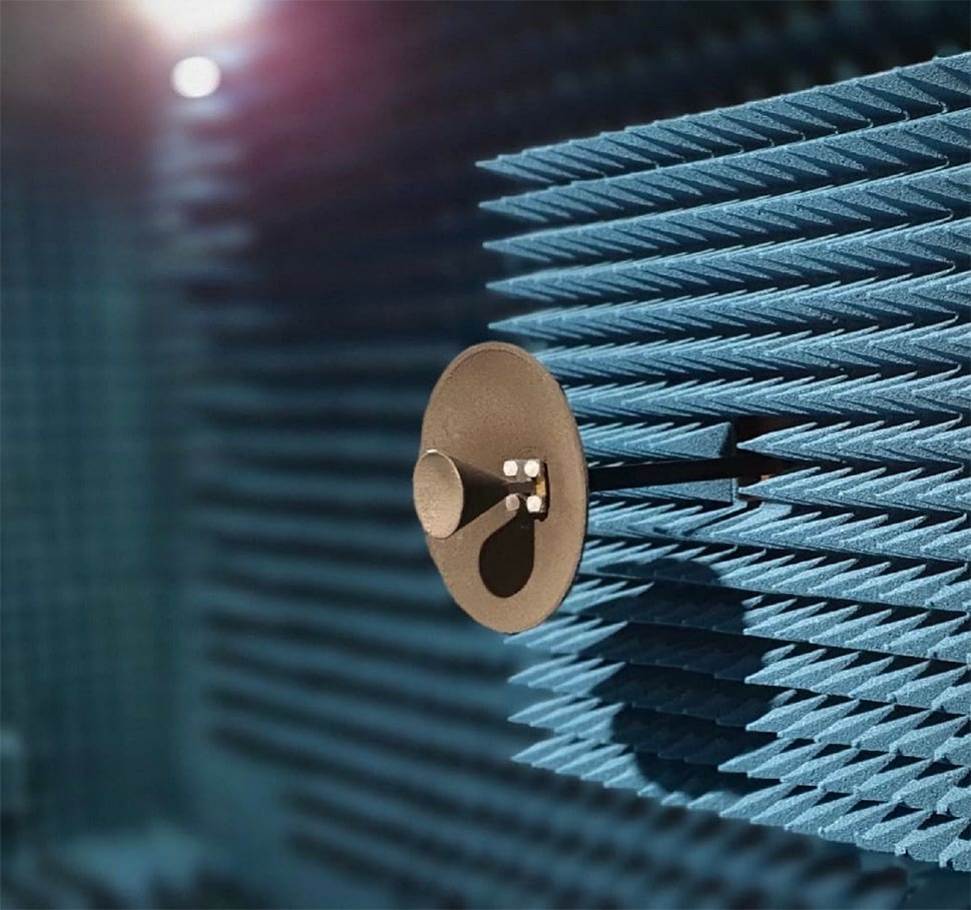
Manufactured wideband mm-Wave antenna in the anechoic chamber at the University of Oviedo.

**Figure 7:**
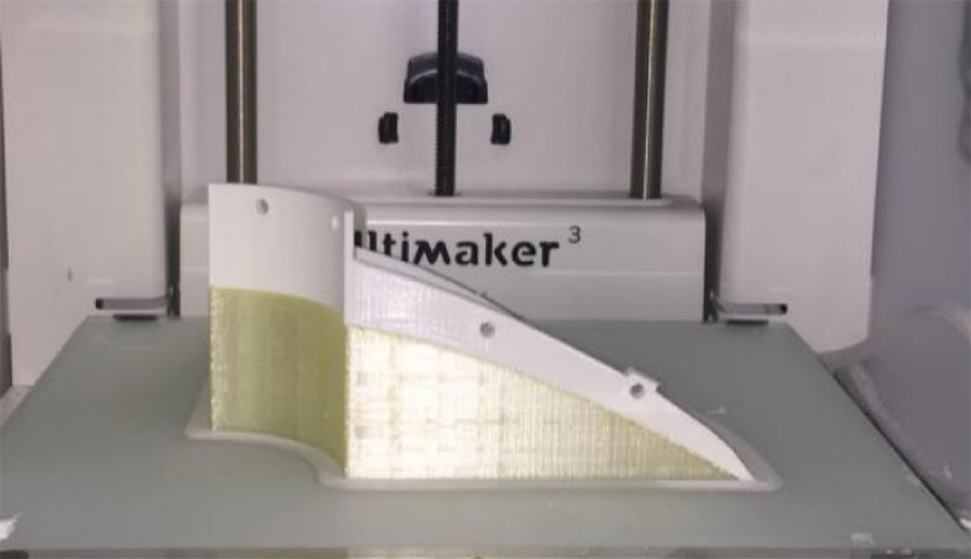
Manufacturing process test by using a sample consisting in a quarter of a parabolic reflector printed in PLA using PVA as supporting structure.

The antenna defined by the geometry of Table 1 has been manufactured to validate its performance. The prototype has been fabricated using the Fused Deposition Modeling (FDM), a 3-D printing technique based on the melting and extrusion of the thermoplastic polymer through a nozzle tup to deposit the material layer-by-layer. As mentioned in the previous section, the thermoplastic used was PLA. Owing to the size of the main reflector ($107.1 \mathrm{mm}$ or $10\lambda @28 \mathrm{GHz}$), it was feasible to manufacture the whole reflector in a single piece, as well as the feeding subsystem (DRW plus hyperbolic subreflector). Then, a conductive spray coating, especially MG 841AR from MG Chemicals^22^, was applied to the hyperbolic (subreflector) and parabolic (main reflector) surfaces to create a reflecting surface. The manufactured and assembled antenna, after coating it, is shown in Fig. 6 in the anechoic chamber. Moreover, Fig. 7 shows a test of the manufacturing process, in which a quarter of a main reflector used as sample is printed using PVA as supporting structure. Then, the PVA is removed with water. The final prototype was monolithically printed using PVA as supporting structure.

### Measured radiation pattern and gain

**Figure 8:**
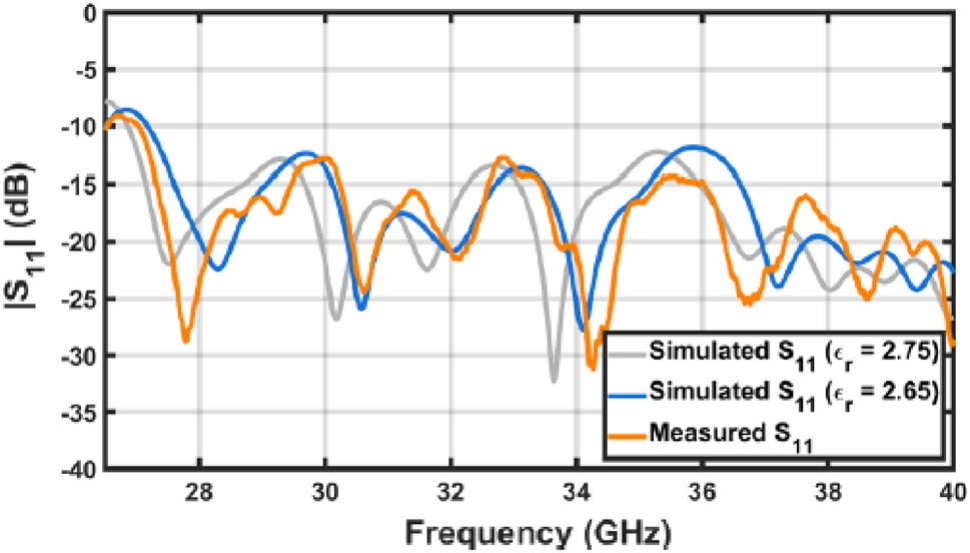
Measured return loss of the manufactured antenna compared with simulations.

The manufactured wideband antenna was measured at facilities of the University of Oviedo to evaluate its performance and compare the results with the full-wave simulations. First, the return loss of the antenna was analyzed over Ka-band. The results of Fig. 8 exhibit a very good agreement between simulations and measurements, as well as a good input matching of the structure with a ${s}_{11}$ below $-13 \mathrm{dB}$ within the whole band, considering the centered optics are considered in the antenna design. Better concordance was obtained by setting the permittivity value to $2.65$, which corrects the slight frequency shift obtained when using the value provided in^21^.

**Figure 9:**
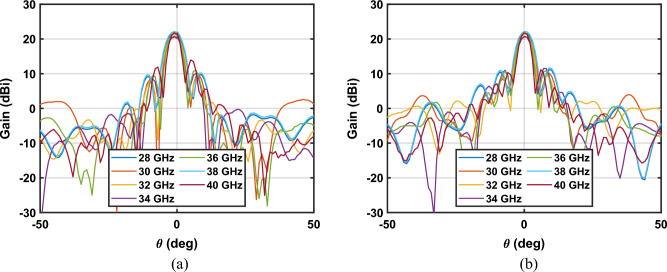
Measured gain at several frequencies in the main planes (**a**) $\phi =0^\circ$ (**b**) $\phi =90^\circ$.

Then, the antenna was measured in the anechoic chamber to get its radiation pattern, Fig. 6. Considering broadband performance shown in return loss measurements, the radiation pattern is measured in the entire Ka-band, which ensures covering the FR2 band centered at $28$ and $39 \mathrm{GHz}$. The setup consisted of a Rohde & Schwarz R&S©ZVK vector network analyzer (VNA) together with the 3D-printed antenna (Antenna Under Test, AUT) and a Flann-Microwave standard pyramid horn of $24.45 \mathrm{dBi}$ gain as probe, both connected to the VNA. The measured patterns at seven frequencies and in the main planes of the antenna ($E$- and $H$- planes) are shown in Fig. 9.

First, the antenna performance is evaluated at the centered frequencies of the 5G-FR2 band, $28$ and $39 \mathrm{GHz}$. The peak gain at the designing frequency ($28 \mathrm{GHz}$) is $21.7 \mathrm{dBi}$, with a SLL of $10.5 \mathrm{dB}$ and a $CP/XP$ of $17.0 \mathrm{dB}$. For the other centered frequency ($39 \mathrm{GHz}$), the measured peak gain is $22.25 \mathrm{dBi}$, the SLL is $11.97 \mathrm{dB}$, and the $CP/XP$ is $17.0$. The $CP/XP$ is evaluated for the cut $\phi =45^\circ$.

In addition to dual-band performance, an overall good agreement between simulations and measurements is obtained, and the antenna exhibits stability in beamwidth, gain and pointing direction for the entire Ka-band, see Fig. 9. For a better evaluation and discussion of the experimental validation of the antenna, the measurements and simulation results at seven frequencies are summarized in Table 2. The difference between the measured and simulated gain is lower than $0.9 \mathrm{dB}$ in the whole band. In the case of side lobes, the disagreement is slightly lager in some cases but in general, the simulated value is obtained for less than $1.2 \mathrm{dB}$. Moreover, the antenna is matched in Ka-band obtaining slight variations in the gain, therefore, the antenna shows a fractional bandwidth of $48.2\%$ when $28 \mathrm{GHz}$ is taken as the central frequency. This approach constituted a broadband solution, and the operational bandwidth of the antenna is only limited by the WR28 single-mode bandwidth.

**Table 2:** Comparison of measurements and simulations.

Freq.	Sim	Meas	Sim	Meas	Sim	Meas	Sim	Meas	Sim	Meas	Sim	Meas	Sim	Meas
	28 GHz		30 GHz		32 GHz		34 GHz		36 GHz		38 GHz		40 GHz	
Gain (dBi)	22.5	21.7	20.5	20.6	22.2	21.4	21	20.8	21	21.8	22.3	22.2	22.1	21.8
SSL (dB)	13.8	10.5	9.3	10.3	10.5	12.1	9.2	9.3	10.5	10.8	11.4	10.5	7.6	7.8
CP/XP *(dB)	21.6	17.0	19.70	21.3	20.08	17.5	18.56	15.5	17.51	19.7	20.48	17.8	20.12	16.8

*The CP/XP is evaluated in the cut $\phi =45^\circ$.

**Figure 10:**
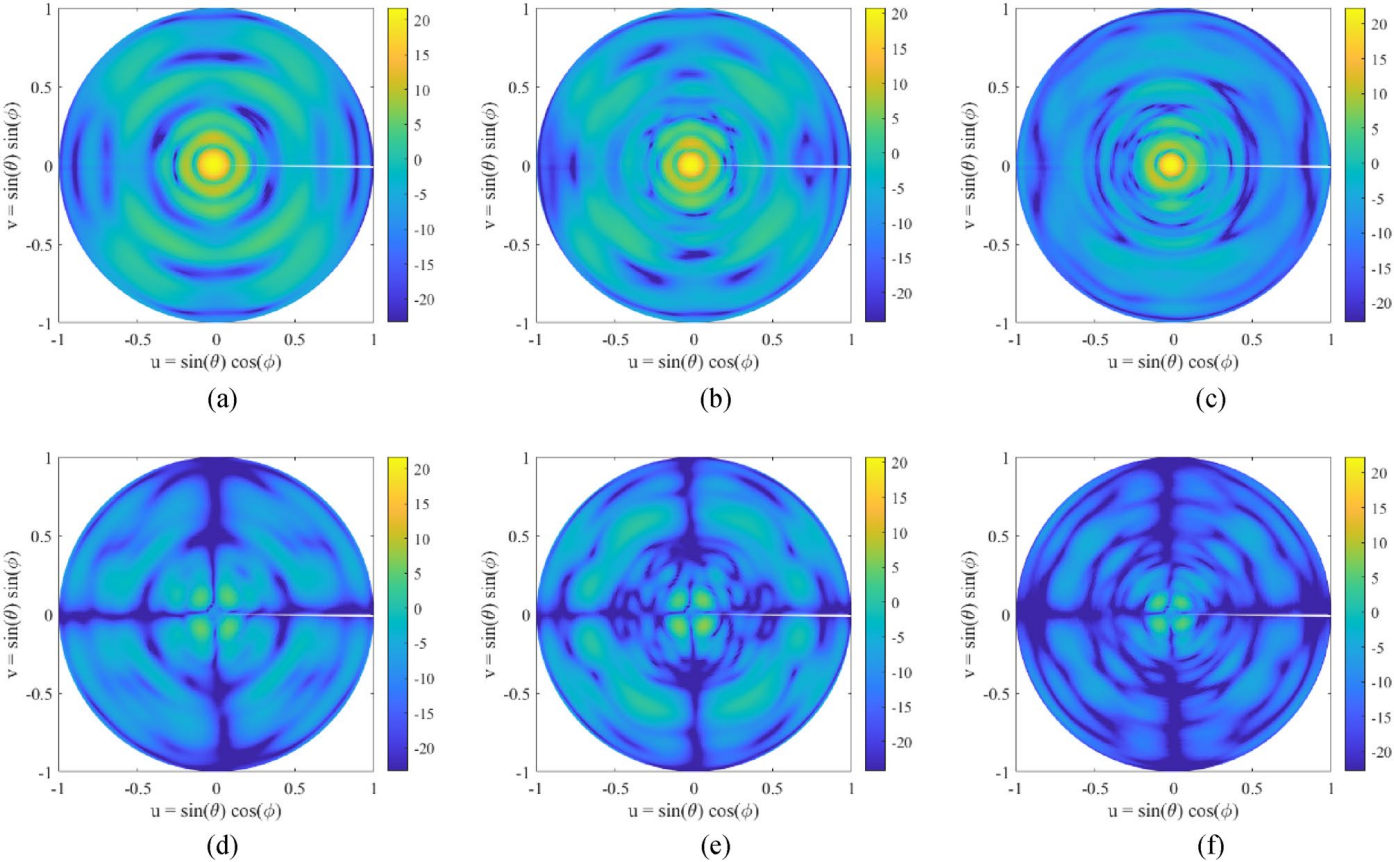
3D measured radiation pattern at several frequencies: $28 \mathrm{GHz}$ (**a**,**d**), $34 \mathrm{GHz}$ (**b**,**e**) and $39 \mathrm{GHz}$ (**c**,**f**) for the copolar (**a**–**c**) and cross-polar component (**d**–**f**).

The 3D radiation pattern has been also measured in the anechoic chamber from $28$ to $40 \mathrm{GHz}$. Figure 10 shows the measurements for three frequencies: $28, 34$ and $39 \mathrm{GHz}$, and both copolar and cross-polar component. The three patterns present a quite similar response, resulting in a well-collimated beam in boresight with a low level of SLL. The detailed side lobes in the main cuts are also shown in Fig. 9, however, in this representation it is observed that this level is kept through the whole radiation pattern. The copolar to crosspolar ration $(CP/XP)$ is over $15 \mathrm{dB}$, as it was concluded from the main cuts through the whole diagram. Besides, the $XP$ reaches a low level with symmetrical distribution, barely changing from one frequency to the other.

**Figure 11:**
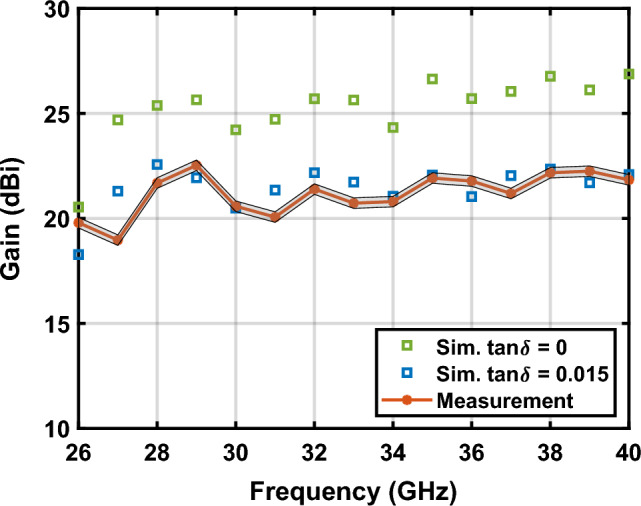
Measured and simulated gain in the entire Ka-band and evaluation of the impact of the dielectric loss. The measured gain considers an error of $\pm 0.25 \mathrm{dB}$ due to the tolerance on the horn antenna used as reference.

The antenna gain has been simulated considering lossy and lossless conditions for PLA to evaluate the impact of using this material at mm-Wave frequencies. However, the metallic surfaces are defined as PEC and therefore this effect is not considered in the simulations. As shown in Fig. 11, the difference in simulated gain for lossy and lossless PLA is $4 \mathrm{dB}$, but the measured gain highly agrees with simulations of lossy PLA cases and lossless metal coating. In the light of these results, the reduction in gain is produced mainly by dielectric loss but not by the limited conductivity of the metallic coating.

## Conclusion

A 3D-printed wideband antenna operating within Ka-band covering the FR2 band is proposed in this work to exploit the increasing interest in mm-Wave communications. A technique based on Cassegrain optics is applied to design the antenna at $28 \mathrm{GHz}$, using a dielectric-only structure to support the reflector and feed the system. Although being traditionally used at lower frequencies with fewer losses, PLA and nickel spray coating are used as low-cost materials to manufacture the antenna with a simple 3D-printing technique and validate the design technique. The high agreement between simulations and measurements at the anechoic chamber reveals the good antenna performance and thus validates potential use of these materials and design technique at mm-Wave frequencies reducing manufacturing complexity and cost. Despite designing the antenna at $28 \mathrm{GHz}$, the higher directivity at $39 \mathrm{GHz}$ compensates the defocused effect produced due to the frequency change, reaching a wideband operation since similar gain (around $22 \mathrm{dB}$) is obtained within the desired band. Moreover, there is antenna matching and little gain variation over Ka-band, resulting in $48.2\%$ of bandwidth with good antenna parameters. These characteristics make the antenna an inexpensive, lightweight, ultra-compact and broadband solution suitable for being used in mm-Wave applications, such as onboard antennas in small platforms.

## Data Availability

The datasets used and/or analyzed during the current study are available from the corresponding author on reasonable request.

## References

[1] Kamoda H, Iwasaki T, Tsumochi J, Kuki T, Hashimoto O (2021). 60-GHz electronically reconfigurable large reflectarray using single-bit phase shiftrs. IEEE Trans. Antennas Propag..

[2] Buffi A, Serra AA, Nepa P, Chou HT, Manara G (2010). A focused planar microstrip array for 2.4 GHz RFID readers. IEEE Trans. Antennas Propag..

[3] Rappaport TS (2013). Millimeter wave mobile communications for 5G cellular: It will work!. IEEE Access.

[4] Darwish T, Kurt GK, Yanikomeroglu H, Bellemare M, Lamontagne G (2022). LEO satellites in 5G and beyond networks: A review from a standardization perspective. IEEE Access.

[5] You L, Li K-X, Wang J, Gao X, Xia X-G, Ottersten B (2020). Massive MIMO transmission for LEO satellite communications. IEEE J. Sel. Areas Commun..

[6] Blackwell, W. J., Eshbaugh, J., Erickson, N. R. and Bryerton, E. Development and flight qualification of the millimeterwave receivers for the NASA TROPICS CubeSat constellation mission. In *2019 44th International Conference on Infrared, Millimeter, and Terahertz Waves (IRMMW-THz)*, pp. 1–1 (2019).

[7] Chahat N, Sauder J, Mitchell M, Beidleman N, Freebury G (2020). One-meter deployable mesh reflector for deep-space network telecommunication at X-band and Ka-band. IEEE Trans. Antennas Propag..

[8] Yurduseven O, Khalily M, Podilchak S, Chattopadhyay G, Fonseca N (2021). Guest editorial: Special cluster on recent advances in antennas for earth and planetary science. IEEE Antennas Wirel. Propag. Lett..

[9] Rao SK (2015). Advanced antenna technologies for satellite communications payloads. IEEE Trans. Antennas Propag..

[10] Mercader-Pellicer S, Tang W, Bresciani D, Legay H, Fonseca NJG, Goussetis G (2021). Angularly stable linear-to-circular polarizing reflectors for multiple beam antennas. IEEE Trans. Antennas Propag..

[11] Sharma SK, Rao S, Shafai L, Sharma SK, Rao S, Shafai L (2013). Handbook of reflector antennas and feed systems volume I: Theory and design of reflectors. Antennas and Propagation.

[12] Kildal P-S (1987). The hat feed: A dual-mode rear-radiating waveguide antenna having low cross polarization. IEEE Trans. Antennaas Propag..

[13] Greco, F., Amendola, G., Boccia, L. and Arnieri, E. A dual band hat feed for reflector antennas in Q-V band. In *Proc. 10th European Conference on Antennas and Propagation (EuCAP)*, pp 1–4 (2016).

[14] Geterud EG, Yang J, Ostling T, Bergmark P (2013). Design and optimization of a compact wideband hat-fed reflector antenna for satellite communications. IEEE Trans. Antennas Propag..

[15] Van der Vorst, M. and Gumpinger, J. Applicability of 3D printing technique for compact Ku-band medium/high-gain antennas. In *2016 10th European Conference on Antennas and Propagation (EuCAP)*, pp. 1–4 (2016).

[16] Sommer, A., Schinagl-Weiβ, A., Hartwanger, C., Kilian, M. and Schneider, M. Multiple spot beam reflector antenna for high throughput satellites using additive manufacturing technology. In *2019 13th European Conference on Antennas and Propagation (EuCAP)*, pp 1-5 (2019).

[17] Rojas-Nastrucci EA, Nussbaum JT, Crane NB, Weller TM (2017). Ka-band characterization of binder jetting for 3-D printing of metallic rectangular waveguide circuits and antennas. IEEE Trans. Microw. Theory Tech..

[18] Romeu J, Blanch S, Vidal N, Lopez-Villegas JM, Aguasca A (2018). Assessment of 3-D printing technologies for millimeter-wave reflectors. IEEE Antennas Wirel. Propag. Lett..

[19] Rebollo A, Vaquero ÁF, Arrebola M, Pino MR (2020). 3D-printed dual-reflector antenna with self-supported dielectric subreflector. IEEE Access.

[20] Felicio, J. M., Fernandes, C. A. and Costa, J. R. Complex permittivity and anisotropy measurement of 3D-printed PLA at microwaves and millimeter-waves. In *Proc. 22nd Int. Conference Applied Electromagnetics and Communications (ICECOM)*, pp. 1–6 (2016).

[21] Systémes, Dassault. *CST Microwave Studio*. http://www.cst.com (Accessed 18 February 2023) (2021).

[22] *841AR Aerosol Datasheet*, MG Chemicals. https://www.mgchemicals.com/downloads/tds/tds-841ar-a.pdf (Accessed February 2023).

